# ADHERE CART versus GWTG-HF for 30-day mortality and intensive care outcomes in emergency department patients with heart failure: A retrospective cohort study (MIMIC-IV-ED)

**DOI:** 10.1097/MD.0000000000049037

**Published:** 2026-05-22

**Authors:** Ahmet Aykut, Cem Yildirim, Mehmet Veysel Öncül, Ertuğ Günsoy, Mehmet Tatli, Ömer Kümet, Remzi Sarikaya

**Affiliations:** a Department of Emergency Medicine, SBU Van Education and Research Hospital, Suphan District, Edremit, Van, Türkiye; b Department of Cardiology, SBU Van Education and Research Hospital, Suphan District, Edremit, Van, Türkiye.

**Keywords:** ADHERE CART, emergency department, GWTG-HF, heart failure, risk stratification

## Abstract

Early risk stratification may support emergency department (ED) decision-making for patients hospitalized with heart failure (HF), yet commonly used tools may perform differently across clinically relevant outcomes. We conducted a retrospective observational cohort study using MIMIC-IV (v3.1) linked to MIMIC-IV-ED (2011–2019). Adult ED encounters with HF International Classification of Diseases-9/10 codes from the ED diagnosis table that were linkable to an inpatient admission were included. The primary analytic cohort was restricted to complete-case encounters with sufficient data to compute both Acute Decompensated Heart Failure National Registry (ADHERE) CART and Get With The Guidelines–Heart Failure (GWTG-HF). The primary outcome was 30-day all-cause mortality; secondary outcomes were intensive care unit (ICU) admission within 24 hours after ED disposition, any ICU admission, acute kidney injury (AKI; Kidney Disease Improving Global Outcomes creatinine criteria), and hospital/ICU length of stay. Associations were evaluated using univariable logistic regression. Discrimination was assessed by area under the receiver operating characteristic curve (bootstrap 95% confidence intervals [CI]s) and compared using DeLong test. Calibration was assessed with patient-level 10-fold cross-validated calibration intercept/slope and calibration curves. Among 5508 eligible encounters, 4812 had complete data for both scores and constituted the analytic cohort. Thirty-day mortality occurred in 317/4812 (6.6%). ICU admission within 24 hours after ED disposition occurred in 822/4812 (17.1%), any ICU admission in 1098/4812 (22.8%), and AKI in 1373/4810 (28.5%). GWTG-HF was associated with higher 30-day mortality (odds ratio 2.3 per 10 points, 95% CI 2.1–2.6); ADHERE CART also showed higher odds across strata (group 4 vs 1 odds ratio 5.2, 95% CI 3.1–8.8). Mortality discrimination was higher for GWTG-HF than ADHERE (area under the receiver operating characteristic curve 0.75 vs 0.64; difference 0.10; *P* < .001), with near-ideal calibration for both. In ED patients hospitalized with HF, GWTG-HF more reliably stratified 30-day mortality risk than ADHERE CART, while both scores showed limited utility for ICU utilization and AKI, supporting outcome-specific and dynamic risk assessment.

## 1. Introduction

Heart failure (HF) remains a leading cause of morbidity and mortality worldwide, and acute HF is a frequent emergency department (ED) presentation that commonly triggers hospital admission and early risk-sensitive decisions regarding monitoring intensity and disposition. Contemporary guidelines and consensus documents emphasize structured, evidence-informed risk assessment in acute HF to support patient-centered decision-making, safe discharge when appropriate, and timely escalation for higher-risk patients.^[1–3]^

Multiple ED-oriented risk tools have been developed to stratify short-term adverse outcomes in acute HF, including instruments designed to identify patients at sufficiently low risk for early events (e.g., STRATIFY) and multivariable scores that estimate short-term mortality risk (e.g., MEESSI). Systematic syntheses highlight substantial heterogeneity across available risk scores in predictors, intended use cases, and external validation, underscoring the need to assess performance in the specific clinical context and endpoint of interest, with attention to both discrimination and calibration.^[4–6]^

Large, openly accessible de-identified electronic health record resources enable reproducible evaluation of established risk stratification approaches in contemporary ED-to-inpatient cohorts. Using the MIMIC-IV database linked to the MIMIC-IV-ED module, we sought to compare 2 routinely calculable HF risk stratification tools, Acute Decompensated Heart Failure National Registry (ADHERE) CART categories and the Get With The Guidelines–Heart Failure (GWTG-HF) score, among adult ED encounters linked to hospitalization. The primary outcome was 30-day all-cause mortality, and secondary outcomes included intensive care unit (ICU) utilization, acute kidney injury (AKI), and length of stay (LOS) outcomes. Model performance was evaluated using association measures, area under the receiver operating characteristic curve (AUROC), and calibration.^[7,8]^ We prespecified that the GWTG-HF score would demonstrate higher discrimination for 30-day mortality than ADHERE CART, while calibration would be assessed for both tools.

## 2. Methods

### 2.1. Study design and time period

This retrospective observational cohort study used the MIMIC-IV, v3.1 database linked to the MIMIC-IV-ED, v2.2 module.^[7–9]^ MIMIC-IV-ED includes ED encounters from 2011 to 2019.^[8]^ The unit of analysis was the ED encounter linked to a subsequent inpatient admission. Eligible encounters occurred between 2011 and 2019, and outcomes were ascertained from linked ED, hospitalization, ICU, laboratory, and mortality records. Follow-up for the primary outcome was defined as 30 days from the index hospital admission time.

### 2.2. Study setting

MIMIC-IV and MIMIC-IV-ED contain de-identified electronic health record data from Beth Israel Deaconess Medical Center, a tertiary academic hospital.^[7–9]^ The databases include time-stamped ED triage measurements, ED vital signs, laboratory measurements, hospital and ICU utilization records, and mortality information required for outcome ascertainment.^[7–9]^

### 2.3. Population

We identified adult ED encounters with a HF-related diagnosis from the MIMIC-IV-ED diagnosis table. HF was defined using any listed ED diagnosis code consistent with HF, rather than restricting the definition to the first-listed or primary diagnosis. The International Classification of Diseases (ICD) code families used for cohort identification were ICD-9 428.* and ICD-10 I50.*. Encounters were then restricted to those linked to an inpatient hospitalization by a non-missing hadm_id in the ED stays table. The complete ICD code list used for cohort identification is provided in Table S1.

The ED encounter, patient, and hospitalization identifiers were stay_id, subject_id, and hadm_id, respectively. The analytic unit was the ED encounter. Therefore, a single patient could contribute more than one eligible encounter if multiple ED presentations met the inclusion criteria during the study period. Repeated encounters were quantified using subject_id, and a sensitivity analysis was performed by restricting the cohort to the first eligible encounter per patient.

The primary analytic cohort was constructed as a complete-case cohort requiring availability of both the ADHERE CART category and the GWTG-HF score. Encounters with missing data preventing computation of either score were excluded to allow paired comparison of both risk tools within the same encounter set.

### 2.4. Outcome measures

The primary outcome was 30-day all-cause mortality, defined as death within 30 days from the index hospital admission time using the death date information available in MIMIC.

Secondary outcomes were ICU admission within 24 hours after ED disposition, any ICU admission during hospitalization, ICU LOS, hospital LOS, and AKI during hospitalization. ICU utilization was identified from the MIMIC-IV ICU icustays table and linked to the index hospitalization using hadm_id. Any ICU admission was defined as the presence of at least one ICU stay during the linked hospitalization. Early ICU admission was defined as the first ICU intime occurring within 24 hours after ED outtime, reflecting ICU transfer shortly after ED disposition. ICU LOS was calculated as cumulative ICU stay duration during the linked hospitalization.

AKI was defined using Kidney Disease: Improving Global Outcomes (KDIGO) 2012 creatinine criteria: an increase in serum creatinine by ≥ 0.3 mg/dL within 48 hours or an increase to ≥ 1.5 times baseline presumed to have occurred within 7 days.^[10]^ Urine output criteria were not used because urine output data were not consistently available for the ED-to-hospital cohort. Baseline creatinine was operationalized as the first available creatinine value within the early index encounter laboratory window, and AKI was coded as KDIGO stage ≥ 1 based on the maximum creatinine rise observed within the 48-hour and 7-day follow-up windows.

### 2.5. Variable extraction and risk score implementation

Systolic blood pressure and heart rate were preferentially extracted from the ED triage table. If triage values were unavailable, the first recorded ED vital sign within 2 hours after ED arrival was used. Laboratory predictors required for the risk scores, including blood urea nitrogen (BUN), creatinine, and sodium, were extracted from time-stamped hospital laboratory records linked by hadm_id. The earliest available value within 24 hours after ED arrival was used for each laboratory component. Laboratory item extraction used predefined candidate laboratory labels and item identifiers, with analyte-specific preference rules when multiple item identifiers were available.

ADHERE CART was implemented as a 4-level ordinal risk category using BUN, systolic blood pressure, and creatinine, following the original derivation report.^[11]^ Group 1 was defined as BUN < 43 mg/dL and systolic blood pressure ≥ 115 mm Hg. Group 2 was defined as either BUN < 43 mg/dL with systolic blood pressure < 115 mm Hg or BUN ≥ 43 mg/dL with systolic blood pressure ≥ 115 mm Hg. Group 3 was defined as BUN ≥ 43 mg/dL, systolic blood pressure < 115 mm Hg, and creatinine < 2.75 mg/dL. Group 4 was defined as BUN ≥ 43 mg/dL, systolic blood pressure < 115 mm Hg, and creatinine ≥ 2.75 mg/dL.

The GWTG-HF score was computed from 7 components: age, systolic blood pressure, heart rate, serum sodium, BUN, chronic obstructive pulmonary disease (COPD) history, and Black race indicator, according to the original score specification.^[12]^ Age at admission was calculated using the MIMIC anchor-age method as anchor age plus the difference between admission year and anchor year. COPD history was identified from hospitalization-level ICD diagnosis codes using ICD-10 J44.* and ICD-9 491, *492*, and 496. The Black race indicator was derived from the admission race field. All 7 GWTG-HF components were required for score calculation; otherwise, the score was coded as missing. GWTG-HF risk categories were also derived according to the original risk-score categories.

Detailed score implementation rules, including ADHERE CART cut-points, GWTG-HF point assignments, and GWTG-HF risk buckets, are provided in Table S2.

### 2.6. Statistical analysis

Continuous variables were summarized as median (interquartile range [IQR]) and categorical variables as n (%). The association of each score with binary outcomes was evaluated using univariable logistic regression models with the risk score as the sole predictor. No additional covariates were included in the primary models because the objective was to compare the prognostic performance of the 2 scores as standalone risk stratification tools. GWTG-HF was analyzed as a continuous score, with effect estimates reported per 10-point increase. ADHERE CART was analyzed as a categorical predictor with group 1 as the reference category for regression-based estimates. For discrimination analyses, ADHERE CART was evaluated using its ordinal group values.

Discrimination was quantified using the AUROC, and 95% confidence intervals were obtained using bootstrap resampling. AUROCs for the 2 score-based models were compared using DeLong test. Calibration was assessed using calibration intercept and slope derived from patient-level 10-fold cross-validated predicted probabilities. Cross-validation folds were constructed using subject_id, ensuring that all encounters from the same patient were assigned to the same fold to avoid leakage across training and validation sets. Calibration curves were constructed by plotting observed outcome frequencies against mean predicted probabilities across deciles of predicted risk.

LOS outcomes were analyzed using generalized linear models suitable for right-skewed distributions, with a Gamma family and log link. Effect estimates were reported as ratios with 95% confidence intervals. ICU LOS analyses were restricted to encounters with any ICU admission.

Missing data were handled using complete-case analysis restricted to encounters with all components required to compute both ADHERE CART and GWTG-HF. No imputation was performed. To evaluate potential selection bias from complete-case restriction, baseline characteristics and outcomes were compared between included and excluded encounters. Loss to follow-up was not applicable because outcomes were ascertained from linked electronic health records and mortality fields in MIMIC.

Because patients could contribute more than one eligible encounter, we quantified the number of unique patients and the distribution of encounters per patient. To account for within-patient correlation in regression-based inference, primary mortality logistic regression models were repeated using patient-level cluster-robust standard errors clustered by subject_id. As a sensitivity analysis, the primary mortality analysis was repeated after restricting the dataset to the first eligible encounter per patient.

Data preprocessing and analyses were performed in Python using Pandas, NumPy, DuckDB, scikit-learn, and statsmodels.

### 2.7. Sample size

No a priori sample size calculation was performed. All eligible ED encounters meeting the inclusion criteria were considered, and the primary analyses were conducted in the complete-case cohort for which both risk scores were computable.

### 2.8. Ethics

The creation of the MIMIC-IV and MIMIC-IV-ED research resources was reviewed and approved by the Institutional Review Board at Beth Israel Deaconess Medical Center, which granted a waiver of informed consent for data sharing (MIMIC-IV-ED IRB #2001P001699).^[8,9]^ This study used de-identified data accessed under the PhysioNet credentialed data use framework.^[9]^

## 3. Results

**Figure 1.**
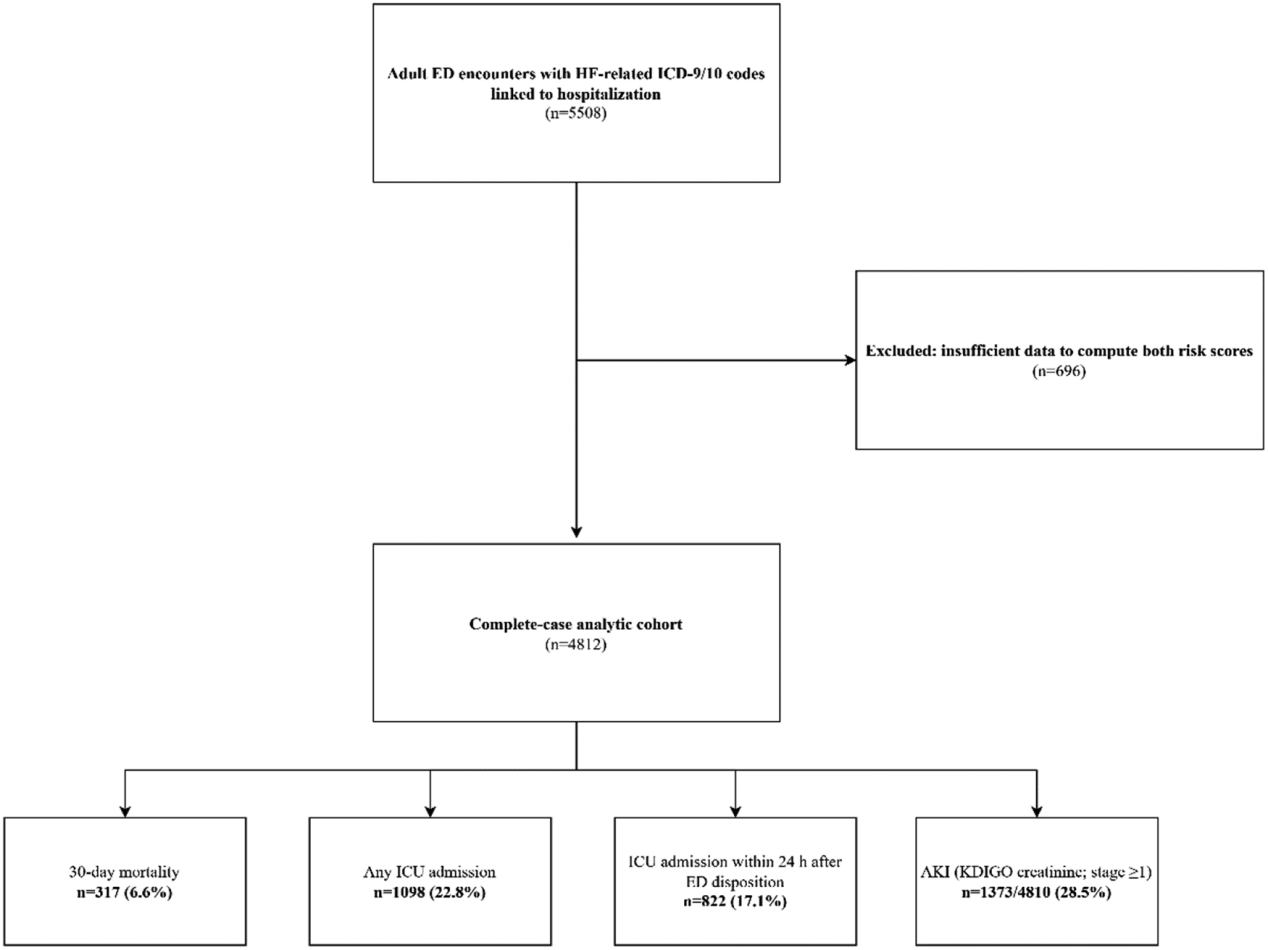
Study flow diagram and outcome frequencies in the analytic cohort. Adult emergency department (ED) encounters with heart failure (HF)-related ICD-9/10 codes (n = 5508) were identified. Encounters with insufficient data to compute both risk scores (ADHERE CART category and GWTG-HF score) were excluded (n = 696), yielding a complete-case analytic cohort (n = 4812). The bottom boxes summarize outcome frequencies within the analytic cohort: 30-day all-cause mortality, any ICU admission, ICU admission within 24 hours, and acute kidney injury (AKI; KDIGO creatinine criteria, stage ≥ 1). ADHERE = Acute Decompensated Heart Failure National Registry, AKI = acute kidney injury, ED = emergency department, GWTG-HF = Get With The Guidelines–Heart Failure, HF = heart failure, ICU = intensive care unit, KDIGO = Kidney Disease: Improving Global Outcomes.

The ICD-based selection rule identified 5508 eligible hospitalized ED encounters with HF, corresponding to 3687 unique patients and 5505 unique hospital admissions. The eligible cohort included 22 distinct HF ICD codes identified from the MIMIC-IV-ED diagnosis table; the complete ICD code list is provided in Table S1. The complete-case analytic dataset included 4812 eligible ED encounters linked to an inpatient admission. These encounters corresponded to 3317 unique patients and 4811 unique hospital admissions. A total of 696 encounters (12.6%) were excluded because either the ADHERE CART category or the GWTG-HF score could not be computed. The primary outcome, 30-day all-cause mortality, occurred in 317/4812 (6.6%). Secondary outcomes included ICU admission within 24 hours after ED disposition in 822/4812 (17.1%), any ICU admission in 1098/4812 (22.8%), and AKI (KDIGO creatinine criteria, stage ≥ 1) in 1373/4810 (28.5%; Fig. 1). Figure 1 summarizes cohort assembly, including the number of eligible encounters identified (n = 5508), the number excluded due to insufficient data to compute both risk scores (n = 696), and the final complete-case analytic cohort (n = 4812). Follow-up for the primary outcome was complete to 30 days using linked mortality records.

Compared with included encounters, excluded encounters had similar age, heart rate, BUN, creatinine, sodium, COPD history, Black race indicator, and 30-day mortality. However, excluded encounters had slightly higher systolic blood pressure, shorter hospital LOS, and higher ICU utilization. Specifically, ICU admission within 24 hours occurred in 174/696 excluded encounters (25.0%) compared with 822/4812 included encounters (17.1%), and any ICU admission occurred in 197/696 excluded encounters (28.3%) compared with 1098/4812 included encounters (22.8%). The missingness profile and included-versus-excluded comparison are provided in Table S3.

Baseline characteristics are summarized in the analytic cohort. Median age was 77 years (IQR 66–86), with triage systolic blood pressure 135 mm Hg (IQR 117–155) and heart rate 82 bpm (IQR 70–96). Early laboratory values showed median urea 10.7 mmol/L (IQR 7.1–17.1), creatinine 124 μmol/L (IQR 88–177), and sodium 140 mmol/L (IQR 136–142). COPD history was present in 949/4812 (19.7%), and Black race in 1125/4812 (23.4%; Table 1). Most baseline variables reported in Table 1 were complete within the analytic cohort. Creatinine-based AKI ascertainment was available for 4810 encounters; therefore, AKI analyses were conducted using this denominator.

**Table 1:** Baseline characteristics of the complete-case analytic cohort (N = 4812).

Characteristic	Overall (N = 4812)
Age, yrs	77 (66–86)
Systolic blood pressure, mm Hg	135 (117–155)
Heart rate, beats/min	82 (70–96)
Urea, mmol/L	10.7 (7.1–17.1)
Creatinine, μmol/L	124 (88–177)
Sodium, mmol/L	140 (136–142)
COPD history, n (%)	949 (19.7%)
Black race, n (%)	1125 (23.4%)
GWTG-HF score	41 (35–48)

Continuous variables are presented as median (IQR) and categorical variables as n (%). Laboratory values are reported in SI units (urea in mmol/L and creatinine in μmol/L).

COPD = chronic obstructive pulmonary disease, GWTG-HF = Get With The Guidelines–Heart Failure, IQR = interquartile range.

Because the unit of analysis was the ED encounter, repeated encounters by the same patient were possible. Of the 3317 unique patients in the final analytic cohort, 2495 (75.2%) contributed 1 encounter, 494 (14.9%) contributed 2 encounters, and 328 (9.9%) contributed 3 or more encounters. Overall, 822 patients (24.8%) contributed more than 1 encounter, accounting for 2317 encounters (48.2% of the analytic cohort). The repeated-encounter distribution is provided in Table S4.

Risk score distributions and crude event rates across strata are shown for both instruments. ADHERE CART categories were: Group 1 55.9%, Group 2 35.9%, Group 3 5.8%, and Group 4 2.3%. Observed 30-day mortality increased across ADHERE groups from 4.0% (Group 1) to 7.9% (Group 2), 18.9% (Group 3), and 17.9% (Group 4). The median GWTG-HF score was 41 (IQR 35–48), with most encounters in the 1% to 5% predicted risk bucket (64.3%) and progressively smaller proportions in higher-risk buckets (each ≤ 1.3% above 15% predicted risk; Table 2).

**Table 2:** Risk score strata and crude outcome frequencies in the analytic cohort (N = 4812).

Risk score	Stratum	n (%)	30-day mortality, n (%)	ICU within 24 h after ED disposition, n (%)	Any ICU, n (%)	AKI, n/N (%)
ADHERE CART	1	2691 (55.9%)	107 (4.0%)	399 (14.8%)	525 (19.5%)	676/2691 (25.1%)
	2	1728 (35.9%)	137 (7.9%)	301 (17.4%)	412 (23.8%)	574/1726 (33.3%)
	3	281 (5.8%)	53 (18.9%)	84 (29.9%)	107 (38.1%)	84/281 (29.9%)
	4	112 (2.3%)	20 (17.9%)	38 (33.9%)	54 (48.2%)	39/112 (34.8%)
GWTG-HF bucket	0–33 (<1%)	917 (19.1%)	12 (1.3%)	121 (13.2%)	165 (18.0%)	277/917 (30.2%)
	34–50 (1–5%)	3093 (64.3%)	155 (5.0%)	497 (16.1%)	667 (21.6%)	845/3091 (27.3%)
	51–57 (>5–10%)	519 (10.8%)	89 (17.1%)	128 (24.7%)	162 (31.2%)	172/519 (33.1%)
	58–61 (>10–15%)	124 (2.6%)	30 (24.2%)	33 (26.6%)	42 (33.9%)	37/124 (29.8%)
	62–65 (>15–20%)	57 (1.2%)	4 (7.0%)	18 (31.6%)	23 (40.4%)	16/57 (28.1%)
	66–70 (>20–30%)	59 (1.2%)	16 (27.1%)	15 (25.4%)	22 (37.3%)	20/59 (33.9%)
	71–74 (>30–40%)	27 (0.6%)	5 (18.5%)	5 (18.5%)	11 (40.7%)	5/27 (18.5%)
	75–78 (>40–50%)	14 (0.3%)	6 (42.9%)	5 (35.7%)	6 (42.9%)	1/14 (7.1%)
	≥79 (>50%)	2 (<0.1%)	0 (0.0%)	0 (0.0%)	0 (0.0%)	0/2 (0.0%)

Distribution of ADHERE CART groups and GWTG-HF risk buckets is shown as n (%). Crude event frequencies are presented for 30-day all-cause mortality, ICU admission within 24 hours, any ICU admission, and AKI. For AKI, denominators reflect non-missing AKI status (n/N, %).

ADHERE = Acute Decompensated Heart Failure National Registry, AKI = acute kidney injury, GWTG-HF = Get With The Guidelines–Heart Failure, ICU = intensive care unit, KDIGO = Kidney Disease: Improving Global Outcomes.

**Figure 2.**
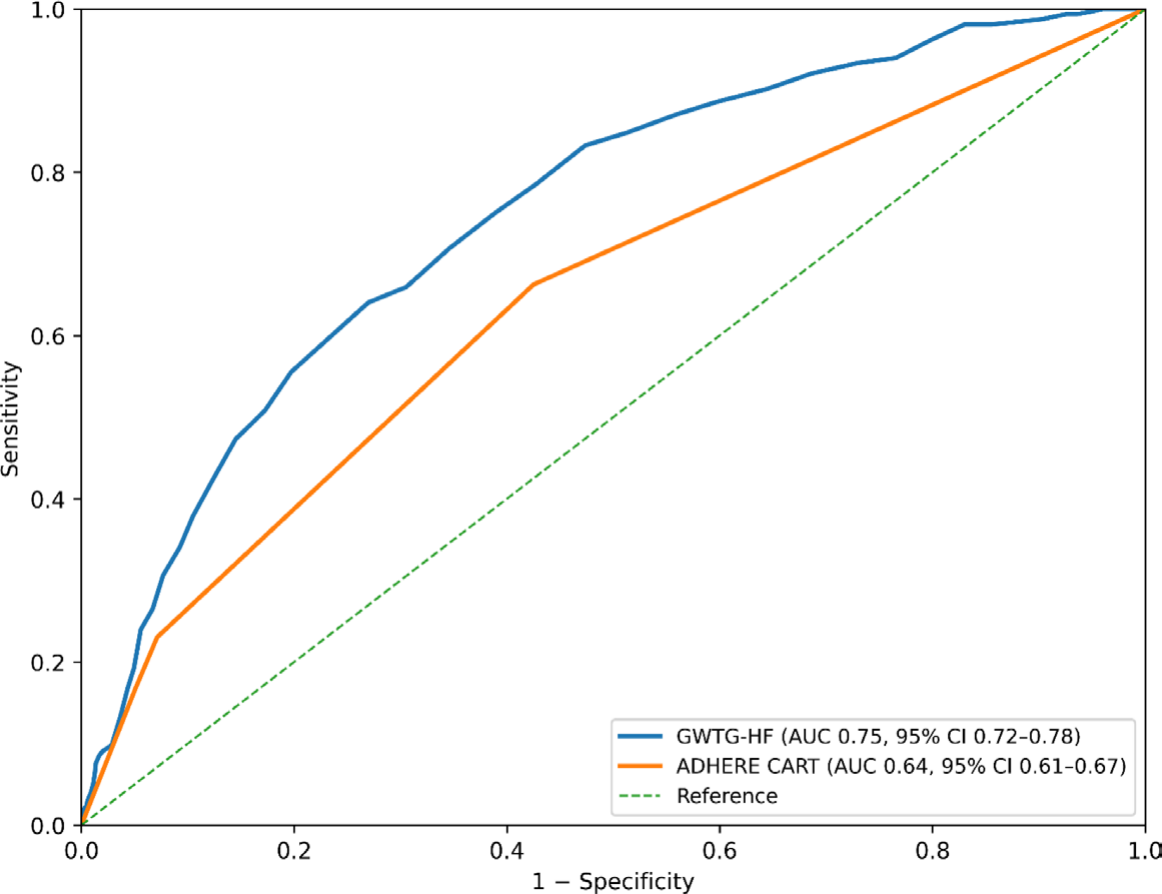
ROC curves for 30-day all-cause mortality. Receiver operating characteristic (ROC) curves comparing discrimination of the GWTG-HF score and ADHERE CART categories for 30-day all-cause mortality. The diagonal dashed line represents no-discrimination (reference). Area under the ROC curve (AUC) values are shown with 95% confidence intervals (bootstrap). AUCs were compared using DeLong test (*P* value reported in Results for the primary outcome comparison). ADHERE, Acute Decompensated Heart Failure National Registry, AUC = area under the curve, CI = confidence interval, GWTG-HF = Get With The Guidelines–Heart Failure, ROC = receiver operating characteristic.

**Figure 3.**
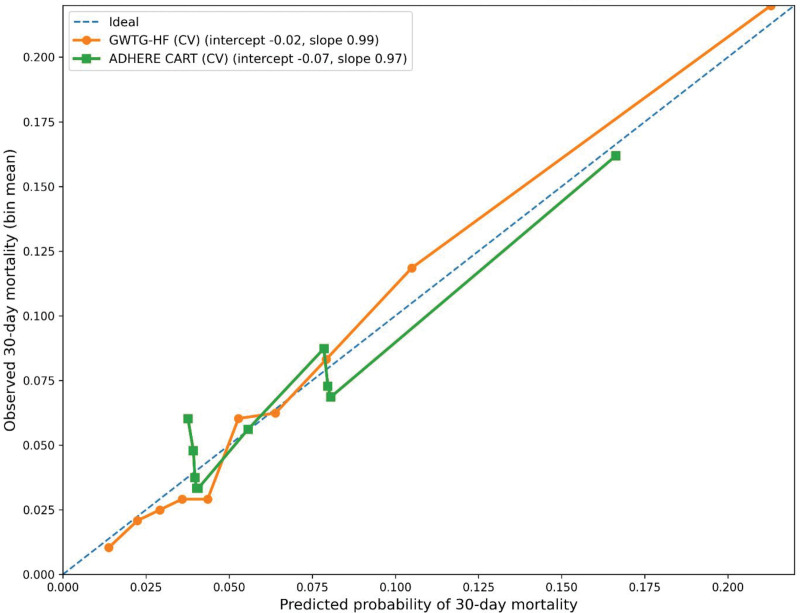
Calibration curves for 30-day mortality (patient-level 10-fold cross-validation). Calibration of predicted 30-day mortality probabilities derived from logistic regression models using GWTG-HF (continuous score) and ADHERE CART (categorical risk groups), evaluated with patient-level 10-fold cross-validation. Points represent observed event rates within deciles of predicted risk, plotted against mean predicted probabilities. The dashed line indicates ideal calibration. Calibration intercepts and slopes shown in the legend correspond to the values reported in Table 3B. ADHERE = Acute Decompensated Heart Failure National Registry, CV = cross-validation, GWTG-HF = Get With The Guidelines–Heart Failure.

For the primary outcome, both scores were associated with 30-day mortality. Per 10-point increase in GWTG-HF score, the odds of 30-day mortality increased (odds ratio [OR] 2.3, 95% confidence interval [CI] 2.1–2.6). Relative to ADHERE Group 1, mortality odds were higher in Group 2 (OR 2.1, 95% CI 1.6–2.7), Group 3 (OR 5.6, 95% CI 3.9–8.0), and Group 4 (OR 5.2, 95% CI 3.1–8.8; Table 3A). Discrimination for 30-day mortality was higher for GWTG-HF (AUROC 0.75, 95% CI 0.72–0.77) than ADHERE (AUROC 0.64, 95% CI 0.62–0.67), with an AUROC difference of 0.10 (DeLong *P* < .001). Calibration was assessed using patient-level 10-fold cross-validation to avoid leakage across repeated encounters from the same patient. Calibration intercepts were close to 0 and slopes close to 1 for both score-based models (GWTG-HF: intercept − 0.02, slope 0.99; ADHERE CART: intercept − 0.07, slope 0.97; Figs. 2 and 3; Table 3B). All reported effect estimates are unadjusted (score-only) models, consistent with the study objective of evaluating scores as standalone tools.

**Table 3:** Primary outcome (30-day all-cause mortality): association, discrimination, and calibration (N = 4812).

(A)			
Model	Contrast		Odds ratio (95% CI)
GWTG-HF	per 10-point increase		2.3 (2.1–2.6)
ADHERE CART	group 2 vs 1		2.1 (1.6–2.7)
ADHERE CART	group 3 vs 1		5.6 (3.9–8.0)
ADHERE CART	group 4 vs 1		5.2 (3.1–8.8)

(B)			
Model	AUROC (95% CI)	Calibration intercept (CV)	Calibration slope (CV)
GWTG-HF	0.75 (0.72–0.77)	−0.02	0.99
ADHERE CART	0.64 (0.61–0.67)	−0.07	0.97
AUROC difference (GWTG-HF − ADHERE CART)	0.10 (DeLong *P* = 8.88 × 10^−16^)	NA	NA

Panel A reports associations between each risk score and 30-day mortality using logistic regression, expressed as odds ratios (ORs) with 95% confidence intervals (CIs). Panel B reports discrimination (AUROC with 95% CIs) and calibration metrics for models using each score. Panel A (Association): For GWTG-HF, the OR is reported per 10-point increase in score. For ADHERE CART, ORs are reported for groups 2 to 4 with group 1 as the reference category. Panel B (Discrimination and calibration): AUROC 95% CIs were estimated using bootstrap resampling. AUROC comparison between GWTG-HF and ADHERE CART used DeLong test; the *P* value is reported only for this primary comparison. Calibration intercept and slope were estimated from 10-fold cross-validated predicted probabilities (intercept ~0 and slope ~1 indicate good calibration).

ADHERE = Acute Decompensated Heart Failure National Registry, AUROC = area under the receiver operating characteristic curve, CI = confidence interval, CV = cross-validation, GWTG-HF = Get With The Guidelines–Heart Failure.

In the first-encounter sensitivity analysis, the cohort was restricted to the first eligible encounter per patient, yielding 3317 unique patients with 229 30-day mortality events (6.9%). The mortality discrimination results were materially unchanged. ADHERE CART had an AUROC of 0.641 (95% CI 0.605–0.679), whereas GWTG-HF had an AUROC of 0.747 (95% confidence interval [CI] 0.717–0.777). The AUROC difference between GWTG-HF and ADHERE CART remained similar to the primary encounter-level analysis (difference, 0.107; 95% CI 0.076–0.138; *P* < .001). These findings are provided in Table S5. Patient-level cluster-robust inference also produced materially unchanged primary mortality associations: GWTG-HF OR 2.32 per 10-point increase (95% CI, 2.08–2.59), ADHERE group 2 versus 1 OR 2.08 (95% CI, 1.60–2.70), group 3 versus 1 OR 5.61 (95% CI, 3.88–8.12), and group 4 versus 1 OR 5.25 (95% CI, 3.14–8.78).

Secondary outcome associations and discrimination are reported across ICU utilization and AKI endpoints. For ICU admission within 24 hours after ED disposition, the GWTG-HF score showed modest association (OR 1.3 per 10 points, 95% CI 1.2–1.4) with AUROC 0.59 (95% CI 0.56–0.61); ADHERE showed AUROC 0.56 (95% CI 0.54–0.58) and higher odds for Group 4 versus Group 1 (OR 2.9, 95% CI 2.0–4.4). Similar patterns were observed for any ICU admission (GWTG-HF: OR 1.4 per 10 points, 95% CI 1.3–1.5; AUROC 0.59, 95% CI 0.57–0.61; ADHERE: AUROC 0.57, 95% CI 0.55–0.59; Group 4 vs Group 1 OR 3.8, 95% CI 2.6–5.6). In contrast, the GWTG-HF score showed no meaningful association with AKI (OR 1.00, 95% CI 0.94–1.07) and poor discrimination (AUROC 0.51, 95% CI 0.49–0.52), whereas ADHERE demonstrated modest AKI discrimination (AUROC 0.55, 95% CI 0.53–0.57; Table 4).

**Table 4:** Secondary binary outcomes: associations and discrimination.

Outcome	GWTG-HF OR per 10 points (95% CI)	GWTG-HF AUROC (95% CI)	ADHERE CART AUROC (95% CI)	ADHERE OR (group 4 vs 1; 95% CI)
ICU admission within 24 h after ED disposition	1.3 (1.2–1.4)	0.59 (0.56–0.61)	0.56 (0.54–0.58)	2.9 (2.0–4.4)
Any ICU admission	1.4 (1.3–1.5)	0.59 (0.57–0.61)	0.57 (0.55–0.59)	3.8 (2.6–5.6)
AKI (KDIGO creatinine; stage ≥ 1)	1.00 (0.94–1.07)	0.51 (0.49–0.52)	0.55 (0.53–0.57)	1.6 (1.1–2.4)

Logistic regression ORs (95% CIs) are reported for each secondary binary outcome (ICU admission within 24 hours, any ICU admission, and AKI): per 10-point increase for GWTG-HF, and (as a summary contrast) group 4 vs group 1 for ADHERE CART. Discrimination is reported as AUROC with 95% CIs (bootstrap).

ADHERE = Acute Decompensated Heart Failure National Registry, AKI = acute kidney injury, AUROC = area under the receiver operating characteristic curve, CI = confidence interval, ED = emergency department, GWTG-HF = Get With The Guidelines–Heart Failure, ICU = intensive care unit, KDIGO = Kidney Disease: Improving Global Outcomes, OR = odds ratio.

LOS outcomes are summarized for hospitalization and ICU subgroups. Median hospital LOS was 5.2 days (IQR 3.0–8.6). In Gamma regression with a log link, each 10-point increase in GWTG-HF score was associated with a 1.1-fold longer hospital stay (95% CI 1.1–1.2). Relative to ADHERE Group 1, hospital LOS was longer in Group 2 (1.2-fold, 95% CI 1.2–1.3), Group 3 (1.5-fold, 95% CI 1.3–1.7), and Group 4 (1.4-fold, 95% CI 1.2–1.7). Among ICU-admitted encounters (n = 1098), median ICU LOS was 2.3 days (IQR 1.2–4.4), and GWTG-HF was associated with longer ICU LOS (1.1-fold per 10 points, 95% CI 1.0–1.2; Table 5).

**Table 5:** Length-of-stay outcomes and regression estimates.

Outcome	Estimate	n
Hospital length of stay, days (median [IQR])	5.2 (3.0–8.6)	4812
Hospital LOS ratio: GWTG-HF (per 10-point increase)	1.1 (1.1–1.2)	
Hospital LOS ratio: ADHERE CART (group 2 vs 1)	1.2 (1.2–1.3)	
Hospital LOS ratio: ADHERE CART (group 3 vs 1)	1.5 (1.3–1.7)	
Hospital LOS ratio: ADHERE CART (group 4 vs 1)	1.4 (1.2–1.7)	
ICU length of stay, days (median [IQR])	2.3 (1.2–4.4)	1098
ICU LOS ratio: GWTG-HF (per 10-point increase)	1.1 (1.0–1.2)	
ICU LOS ratio: ADHERE CART (group 2 vs 1)	1.1 (0.9–1.3)	
ICU LOS ratio: ADHERE CART (group 3 vs 1)	1.1 (0.8–1.4)	
ICU LOS ratio: ADHERE CART (group 4 vs 1)	1.3 (0.9–1.9)	

Hospital and ICU length of stay are presented as median (IQR). Associations with length-of-stay outcomes were analyzed using Gamma regression with a log link; effect estimates are reported as ratios (exponentiated coefficients) with 95% CIs per 10-point increase in GWTG-HF and by ADHERE CART group (2–4 vs 1). ICU LOS analyses are restricted to encounters with any ICU admission.

ADHERE = Acute Decompensated Heart Failure National Registry, CI = confidence interval, GWTG-HF = Get With The Guidelines–Heart Failure, ICU = intensive care unit, IQR = interquartile range, LOS = length of stay.

## 4. Discussion

Across a complete-case cohort of hospitalized ED patients with HF, both ADHERE CART and GWTG-HF demonstrated clinically coherent risk gradients for 30-day mortality, but they behaved differently across downstream endpoints. In line with our objective of comparing score performance for 30-day mortality and intensive care-related outcomes, the key findings were higher discrimination for 30-day mortality with GWTG-HF than ADHERE, near-ideal calibration for both score-based models for mortality, and only modest discrimination for ICU utilization and AKI endpoints, highlighting outcome-specific utility. These findings were robust in a first-encounter sensitivity analysis restricted to 1 encounter per patient, in which GWTG-HF maintained higher discrimination than ADHERE CART for 30-day mortality. The stronger discrimination and near-ideal calibration observed for the GWTG-HF-based model for mortality are consistent with its broader variable set capturing age and physiological reserve in addition to hemodynamic and renal markers, whereas the more limited performance of ADHERE for 30-day mortality likely reflects its intentionally parsimonious structure. These findings support the view that bedside HF risk tools remain informative when transported into EHR-based ED cohorts, but that their utility is outcome-specific (mortality vs escalation of care vs kidney injury) and sensitive to differences between derivation targets and contemporary ED-to-inpatient workflows.^[11,12]^

The present findings should be interpreted within the broader landscape of HF risk stratification. Existing tools differ in target population, time horizon, predictor structure, and intended clinical use. Some scores are designed for early acute-care decisions, others for in-hospital mortality, and others for longer-term prognosis in chronic or device-treated HF populations. Comparative data from high-risk device-treated HF cohorts have similarly suggested that the relative performance of mortality prediction scores may vary according to population and prediction horizon.^[13]^ This context is important because the appropriate score depends not only on statistical performance, but also on whether the intended endpoint is mortality, disposition, escalation of care, renal injury, or longitudinal prognosis.

In the derivation study from ADHERE, the CART approach was designed as a pragmatic triage tool for in-hospital mortality using a small set of readily available variables (BUN, systolic blood pressure, and creatinine), prioritizing bedside usability over maximal discrimination.^[11]^ The GWTG-HF risk score was also developed for in-hospital mortality but incorporated additional clinical predictors, and subsequent validations have reported stable prognostic performance across diverse HF populations and time horizons, including post-admission outcomes in some settings.^[12,14]^ From that perspective, the observed superiority of GWTG-HF for 30-day mortality in the present ED-linked cohort is directionally consistent with prior work suggesting that broader clinical feature sets better sustain discrimination when transported beyond the original endpoint definition and clinical context.^[12,14]^

When compared with ED-specific acute HF risk instruments explicitly optimized for short-term mortality (e.g., EHMRG and MEESSI-AHF), the present results reinforce a recurring theme: models can perform well for mortality yet show weaker performance for nonfatal outcomes such as admission-related events or revisits, which are influenced by local practice patterns, bed availability, and heterogeneous thresholds for ICU-level care.^[5,15,16]^ The modest discrimination observed for ICU-related endpoints is therefore not unexpected and aligns with prior evidence that disposition and escalation outcomes are harder to predict using static presentation-time variables alone, particularly when the decision process is partly operational rather than purely pathophysiological.^[15,17]^ In the present study, early ICU admission was defined relative to ED disposition, which captures early escalation after ED care rather than ICU transfer from the moment of ED arrival. Accordingly, ICU-related performance metrics should be interpreted cautiously as reflecting both patient severity and system-level decision processes.

The limited performance for AKI also deserves specific interpretation. ADHERE includes renal and hemodynamic variables and therefore showed slightly better AKI discrimination than GWTG-HF, but neither score was developed as a kidney-injury prediction tool. In HF, AKI risk reflects dynamic cardiorenal interactions, congestion, diuretic exposure, perfusion pressure, baseline kidney function, and evolving in-hospital treatment response. A single presentation-time score is therefore unlikely to fully capture kidney-related risk. This supports the need for dynamic reassessment using serial renal function and congestion markers rather than relying on static mortality-oriented scores for AKI prediction.

Recent literature also suggests that adjunct biological and metabolic markers may complement traditional risk scores in selected HF populations. For example, systemic immune-inflammation indices and the triglyceride-glucose index have been associated with prognosis in patients with HF with reduced ejection fraction and implantable cardioverter-defibrillators.^[18,19]^ These studies are not directly comparable to an ED acute-care cohort, but they support the broader concept that inflammation, metabolic stress, and immune activation may add prognostic information beyond conventional bedside variables. In ED-to-inpatient HF prediction, such markers should be evaluated prospectively and externally before being incorporated into clinical decision pathways.

Several limitations warrant emphasis. First, this was a retrospective, single-center analysis using de-identified EHR data from MIMIC-IV and MIMIC-IV-ED; local practice patterns and coding conventions may limit generalizability.^[7,8]^ Second, restricting analyses to the complete-case cohort may introduce selection bias if missingness was not random. In this study, 696 of 5508 otherwise eligible encounters (12.6%) were excluded because at least 1 of the 2 scores could not be computed. Included and excluded encounters had similar age, heart rate, BUN, creatinine, sodium, COPD history, Black race indicator, and 30-day mortality, but excluded encounters had higher ICU utilization and shorter hospital LOS. Therefore, selection bias related to differential availability of score components cannot be excluded, particularly for secondary outcomes related to ICU use. Third, both scores were reestimated using logistic models and applied to an endpoint (30-day mortality) different from the original in-hospital derivation target, which can affect transportability.^[11,12]^ Fourth, AKI was defined using KDIGO creatinine criteria without urine output, and baseline creatinine was operationalized using the first available measurement, which may misclassify community-acquired kidney injury or dilute sensitivity to early AKI.^[10]^ Fifth, the unit of analysis was the ED encounter, and some patients contributed more than one encounter. Although repeated encounters accounted for a substantial proportion of the analytic dataset, the first-encounter sensitivity analysis produced materially unchanged mortality discrimination results. Finally, ICU admission outcomes are partly determined by system-level factors and may not reflect uniform severity across patients.

Clinically, these findings suggest that in ED patients hospitalized with HF, GWTG-HF may be preferable for early mortality risk communication and prioritization of monitoring intensity, whereas ADHERE’s emphasis on renal and hemodynamic markers may remain useful as a rapid red flag framework, particularly in contexts where renal vulnerability and perfusion status drive early complications.^[11,12,20]^ Given the known prognostic impact of AKI in HF and the heterogeneity of cardiorenal trajectories during decompensation, clinicians should interpret risk stratification alongside evolving renal function and congestion markers rather than rely on a single presentation-time score for kidney-related outcomes.^[20]^ The results should not be interpreted as supporting fixed score thresholds for ED disposition decisions. Rather, they indicate that score choice should be aligned with the target endpoint and should be integrated with clinical judgment, local admission pathways, and reassessment after initial ED management. Overall, our findings should be interpreted as comparative performance evidence in an ED-to-inpatient cohort rather than as a mandate to replace clinical judgment or local pathways, and external validation is required before adopting fixed thresholds for disposition decisions.

Future research should focus on ED-to-inpatient HF models explicitly trained for multiple clinically meaningful endpoints (mortality, ICU utilization, and AKI), incorporating dynamic updates (serial creatinine, lactate/biomarkers where available, treatment response) and evaluating transportability with external validation across institutions.^[7,8]^ For kidney outcomes, refinement should align with contemporary KDIGO phenotyping discussions and consider complementary definitions beyond creatinine-only criteria when feasible.^[10]^ Future model development should follow contemporary prediction-model reporting principles, including transparent variable definition, internal validation, calibration assessment, external validation, and decision-analytic evaluation.^[21,22]^ Finally, decision-analytic evaluation, such as net benefit across clinically relevant thresholds, would help translate statistical performance into actionable ED pathways and clarify where simple scores are sufficient versus where ED-specific, context-aware models are needed.^[22]^ Because this analysis is based on a single-center US EHR dataset and restricted to hospitalized ED encounters with complete score data, external validity is primarily to similar tertiary-care settings and populations; generalizability to non-US systems, nonhospitalized ED HF presentations, and settings with different ICU admission practices may be limited.

## 5. Conclusion

In this ED-linked hospitalized HF cohort, both ADHERE CART and GWTG-HF provided clinically coherent early risk stratification, but their utility was outcome-specific. GWTG-HF more reliably identified patients at higher short-term mortality risk, and this finding was robust in a first-encounter sensitivity analysis. In contrast, neither tool sufficiently captured escalation-of-care or kidney injury trajectories on its own. Clinically, these findings support the use of readily available presentation-time scores to standardize early mortality risk communication and guide monitoring intensity, while recognizing that ICU use and AKI are shaped by evolving physiology, treatment response, and contextual care processes. These outcomes require dynamic reassessment rather than single-score decision-making.

## Author contributions

**Conceptualization:** Ahmet Aykut.

**Data curation:** Ahmet Aykut.

**Formal analysis:** Ahmet Aykut.

**Investigation:** Cem Yildirim, Mehmet Veysel Öncül, Mehmet Tatli, Ömer Kümet, Remzi Sarikaya.

**Methodology:** Ahmet Aykut, Cem Yildirim, Mehmet Veysel Öncül, Ertuğ Günsoy, Mehmet Tatli, Ömer Kümet, Remzi Sarikaya.

**Project administration:** Ahmet Aykut.

**Software:** Ahmet Aykut.

**Supervision:** Ahmet Aykut, Ertuğ Günsoy.

**Validation:** Ahmet Aykut, Cem Yildirim, Mehmet Veysel Öncül, Ertuğ Günsoy, Mehmet Tatli, Ömer Kümet, Remzi Sarikaya.

**Visualization:** Ahmet Aykut.

**Writing – original draft:** Ahmet Aykut.

**Writing – review & editing:** Ahmet Aykut, Cem Yildirim, Mehmet Veysel Öncül, Ertuğ Günsoy, Mehmet Tatli, Ömer Kümet, Remzi Sarikaya.










