# Supplementary material for: ADHERE CART versus GWTG-HF for 30-day mortality and intensive care outcomes in emergency department patients with heart failure: A retrospective cohort study (MIMIC-IV-ED)
Source: Medicine (Baltimore). 2026 May 22;105(21):e49037. doi: 10.1097/MD.0000000000049037 (PMC13200985; doi:10.1097/MD.0000000000049037)
Supplement: Supplementary file 1 [file medi-105-e49037-s001.docx]

| **Supplementary Table S1. Heart failure ICD codes used to identify eligible ED encounters** |
| --- |
| Heart failure was identified using any listed ED diagnosis code in MIMIC-IV-ED diagnosis.csv.gz, ICD-9 428.* or ICD-10 I50.*, restricted to ED encounters linked to inpatient admission by non-missing hadm_id. |

| **Cohort identification rule** | |
| --- | --- |
| **Item** | **Definition** |
| Diagnosis source | MIMIC-IV-ED diagnosis table (ed/diagnosis.csv.gz) |
| Diagnosis fields | Any listed ED diagnosis code; not restricted to first-listed/primary diagnosis |
| ICD-9 heart failure rule | ICD-9 codes beginning with 428 |
| ICD-10 heart failure rule | ICD-10 codes beginning with I50 |
| Admission linkage | Restricted to ED encounters with non-missing hadm_id in edstays.csv.gz |
| Encounter identifier | stay_id |
| Patient identifier | subject_id |
| Hospital admission identifier | hadm_id |

| **Overall counts** | | | | | |
| --- | --- | --- | --- | --- | --- |
| **Cohort** | **HF diagnosis rows** | **Encounters** | **Unique patients** | **Unique admissions** | **Unique HF ICD codes** |
| Eligible cohort | 5517 | 5508 | 3687 | 5505 | 22 |
| Final complete-case cohort | 4820 | 4812 | 3317 | 4811 | 19 |

| **ICD code counts** | | | | | | | | | | |
| --- | --- | --- | --- | --- | --- | --- | --- | --- | --- | --- |
| **ICD version** | **ICD code** | **Diagnosis title** | **Eligible diagnosis rows** | **Eligible encounters with code** | **Eligible patients with code** | **Eligible admissions with code** | **Final diagnosis rows** | **Final encounters with code** | **Final patients with code** | **Final admissions with code** |
| 9 | 428.0 | Congestive heart failure, unspecified | 2634 | 2634 | 1870 | 2633 | 2343 | 2343 | 1712 | 2342 |
| 9 | 428.1 | Left heart failure | 2 | 2 | 2 | 2 | 2 | 2 | 2 | 2 |
| 9 | 428.2 | Systolic heart failure, unspecified | 1 | 1 | 1 | 1 | 1 | 1 | 1 | 1 |
| 9 | 428.21 | Acute systolic heart failure | 4 | 4 | 4 | 4 | 3 | 3 | 3 | 3 |
| 9 | 428.23 | Acute on chronic systolic heart failure | 2 | 2 | 2 | 2 | 2 | 2 | 2 | 2 |
| 9 | 428.3 | Diastolic heart failure, unspecified | 3 | 3 | 3 | 3 | 3 | 3 | 3 | 3 |
| 9 | 428.31 | Acute diastolic heart failure | 3 | 3 | 3 | 3 | 3 | 3 | 3 | 3 |
| 9 | 428.33 | Acute on chronic diastolic heart failure | 1 | 1 | 1 | 1 | 1 | 1 | 1 | 1 |
| 9 | 428.9 | Heart failure, unspecified | 68 | 68 | 68 | 68 | 65 | 65 | 65 | 65 |
| 10 | I50.1 | Left ventricular failure, unspecified | 1 | 1 | 1 | 1 | 0 | 0 | 0 | 0 |
| 10 | I5020 | Unspecified systolic (congestive) heart failure | 5 | 5 | 5 | 5 | 5 | 5 | 5 | 5 |
| 10 | I50.21 | Acute systolic (congestive) heart failure | 3 | 3 | 3 | 3 | 3 | 3 | 3 | 3 |
| 10 | I50.30 | Unspecified diastolic (congestive) heart failure | 4 | 4 | 4 | 4 | 3 | 3 | 3 | 3 |
| 10 | I50.31 | Acute diastolic (congestive) heart failure | 2 | 2 | 2 | 2 | 2 | 2 | 2 | 2 |
| 10 | I50.32 | Chronic diastolic (congestive) heart failure | 1 | 1 | 1 | 1 | 1 | 1 | 1 | 1 |
| 10 | I50.40 | Unspecified combined systolic (congestive) and diastolic (congestive) heart failure | 28 | 28 | 26 | 27 | 11 | 11 | 11 | 11 |
| 10 | I50.41 | Acute combined systolic (congestive) and diastolic (congestive) heart failure | 15 | 15 | 15 | 15 | 5 | 5 | 5 | 5 |
| 10 | I50.42 | Chronic combined systolic (congestive) and diastolic (congestive) heart failure | 1 | 1 | 1 | 1 | 0 | 0 | 0 | 0 |
| 10 | I50.43 | Acute on chronic combined systolic (congestive) and diastolic (congestive) heart failure | 2 | 2 | 2 | 2 | 0 | 0 | 0 | 0 |
| 10 | I50.810 | Right heart failure, unspecified | 1 | 1 | 1 | 1 | 1 | 1 | 1 | 1 |
| 10 | I50.89 | Other heart failure | 2 | 2 | 2 | 2 | 2 | 2 | 2 | 2 |
| 10 | I50.9 | Heart failure, unspecified | 2734 | 2734 | 2006 | 2733 | 2364 | 2364 | 1781 | 2364 |
