# Supplementary material for: ADHERE CART versus GWTG-HF for 30-day mortality and intensive care outcomes in emergency department patients with heart failure: A retrospective cohort study (MIMIC-IV-ED)
Source: Medicine (Baltimore). 2026 May 22;105(21):e49037. doi: 10.1097/MD.0000000000049037 (PMC13200985; doi:10.1097/MD.0000000000049037)
Supplement: Supplementary file 2 [file medi-105-e49037-s002.docx]

| **Supplementary Table S2. ADHERE CART and GWTG-HF implementation rules** |
| --- |
| Rules reflect the score implementation used in the analysis dataset. GWTG-HF required all 7 components. |

| **A. ADHERE CART category definitions** | | | | |
| --- | --- | --- | --- | --- |
| **Score** | **Component/rule** | **Definition** | **Category or points** | **Notes** |
| ADHERE CART | Group 1 | BUN <43 mg/dL and SBP ≥115 mmHg | Group 1 | Reference/lowest-risk group |
| ADHERE CART | Group 2 | BUN <43 mg/dL and SBP <115 mmHg, or BUN ≥43 mg/dL and SBP ≥115 mmHg | Group 2 |  |
| ADHERE CART | Group 3 | BUN ≥43 mg/dL, SBP <115 mmHg, and creatinine <2.75 mg/dL | Group 3 |  |
| ADHERE CART | Group 4 | BUN ≥43 mg/dL, SBP <115 mmHg, and creatinine ≥2.75 mg/dL | Group 4 | Highest-risk group |

| **B. GWTG-HF point assignment** | | | | |
| --- | --- | --- | --- | --- |
| **Score** | **Component** | **Range or definition** | **Points** | **Notes** |
| GWTG-HF | Age, years | ≤19 | 0 | MIMIC anchor-age method |
| GWTG-HF | Age, years | 20-29 | 3 | MIMIC anchor-age method |
| GWTG-HF | Age, years | 30-39 | 5 | MIMIC anchor-age method |
| GWTG-HF | Age, years | 40-49 | 8 | MIMIC anchor-age method |
| GWTG-HF | Age, years | 50-59 | 11 | MIMIC anchor-age method |
| GWTG-HF | Age, years | 60-69 | 14 | MIMIC anchor-age method |
| GWTG-HF | Age, years | 70-79 | 16 | MIMIC anchor-age method |
| GWTG-HF | Age, years | 80-89 | 19 | MIMIC anchor-age method |
| GWTG-HF | Age, years | 90-99 | 22 | MIMIC anchor-age method |
| GWTG-HF | Age, years | 100-109 | 25 | MIMIC anchor-age method |
| GWTG-HF | Age, years | ≥110 | 27 | MIMIC anchor-age method |
| GWTG-HF | SBP, mmHg | <60 | 28 |  |
| GWTG-HF | SBP, mmHg | 60-69 | 26 |  |
| GWTG-HF | SBP, mmHg | 70-79 | 24 |  |
| GWTG-HF | SBP, mmHg | 80-89 | 23 |  |
| GWTG-HF | SBP, mmHg | 90-99 | 21 |  |
| GWTG-HF | SBP, mmHg | 100-109 | 19 |  |
| GWTG-HF | SBP, mmHg | 110-119 | 17 |  |
| GWTG-HF | SBP, mmHg | 120-129 | 15 |  |
| GWTG-HF | SBP, mmHg | 130-139 | 13 |  |
| GWTG-HF | SBP, mmHg | 140-149 | 11 |  |
| GWTG-HF | SBP, mmHg | 150-159 | 9 |  |
| GWTG-HF | SBP, mmHg | 160-169 | 8 |  |
| GWTG-HF | SBP, mmHg | 170-179 | 6 |  |
| GWTG-HF | SBP, mmHg | 180-189 | 4 |  |
| GWTG-HF | SBP, mmHg | 190-199 | 2 |  |
| GWTG-HF | SBP, mmHg | ≥200 | 0 |  |
| GWTG-HF | Heart rate, bpm | ≤79 | 0 |  |
| GWTG-HF | Heart rate, bpm | 80-84 | 1 |  |
| GWTG-HF | Heart rate, bpm | 85-89 | 3 |  |
| GWTG-HF | Heart rate, bpm | 90-94 | 4 |  |
| GWTG-HF | Heart rate, bpm | 95-99 | 5 |  |
| GWTG-HF | Heart rate, bpm | 100-104 | 6 |  |
| GWTG-HF | Heart rate, bpm | ≥105 | 8 |  |
| GWTG-HF | Serum sodium, mmol/L | ≤130 | 4 | As implemented in analysis code |
| GWTG-HF | Serum sodium, mmol/L | 131-133 | 3 | As implemented in analysis code |
| GWTG-HF | Serum sodium, mmol/L | 134 | 2 | As implemented in analysis code |
| GWTG-HF | Serum sodium, mmol/L | 135-136 | 2 | As implemented in analysis code |
| GWTG-HF | Serum sodium, mmol/L | 137-138 | 1 | As implemented in analysis code |
| GWTG-HF | Serum sodium, mmol/L | ≥139 | 0 | As implemented in analysis code |
| GWTG-HF | BUN, mg/dL | ≤9 | 0 |  |
| GWTG-HF | BUN, mg/dL | 10-19 | 2 |  |
| GWTG-HF | BUN, mg/dL | 20-29 | 4 |  |
| GWTG-HF | BUN, mg/dL | 30-39 | 6 |  |
| GWTG-HF | BUN, mg/dL | 40-49 | 8 |  |
| GWTG-HF | BUN, mg/dL | 50-59 | 9 |  |
| GWTG-HF | BUN, mg/dL | 60-69 | 11 |  |
| GWTG-HF | BUN, mg/dL | 70-79 | 13 |  |
| GWTG-HF | BUN, mg/dL | 80-89 | 15 |  |
| GWTG-HF | BUN, mg/dL | 90-99 | 17 |  |
| GWTG-HF | BUN, mg/dL | 100-109 | 19 |  |
| GWTG-HF | BUN, mg/dL | 110-119 | 21 |  |
| GWTG-HF | BUN, mg/dL | 120-129 | 23 |  |
| GWTG-HF | BUN, mg/dL | 130-139 | 25 |  |
| GWTG-HF | BUN, mg/dL | 140-149 | 27 |  |
| GWTG-HF | BUN, mg/dL | ≥150 | 28 |  |
| GWTG-HF | COPD history | No | 0 | Hospitalization-level ICD codes |
| GWTG-HF | COPD history | Yes | 2 | ICD-10 J44.* or ICD-9 491.*, 492.*, 496 |
| GWTG-HF | Black race indicator | Black | 0 | Derived from admission race field |
| GWTG-HF | Black race indicator | Non-Black | 3 | Derived from admission race field |

| **C. GWTG-HF risk buckets** | |
| --- | --- |
| **GWTG-HF score** | **Estimated in-hospital mortality bucket** |
| 0-33 | <1% |
| 34-50 | 1-5% |
| 51-57 | >5-10% |
| 58-61 | >10-15% |
| 62-65 | >15-20% |
| 66-70 | >20-30% |
| 71-74 | >30-40% |
| 75-78 | >40-50% |
| ≥79 | >50% |
