# Supplementary material for: ADHERE CART versus GWTG-HF for 30-day mortality and intensive care outcomes in emergency department patients with heart failure: A retrospective cohort study (MIMIC-IV-ED)
Source: Medicine (Baltimore). 2026 May 22;105(21):e49037. doi: 10.1097/MD.0000000000049037 (PMC13200985; doi:10.1097/MD.0000000000049037)
Supplement: Supplementary file 3 [file medi-105-e49037-s003.docx]

| **Supplementary Table S3. Missingness profile and included-versus-excluded comparison** |
| --- |
| Complete-case cohort required availability of both ADHERE CART and GWTG-HF. Values are median [IQR] or n/N (%) unless otherwise specified. |

| **A. Included-versus-excluded comparison** | | | |
| --- | --- | --- | --- |
| **Variable** | **Included complete-case cohort (n=4,812)** | **Excluded due to missing score data (n=696)** | **P value** |
| Age, years | 77.00 [66.00-86.00] | 77.00 [66.00-86.00] | 0.521 |
| Systolic blood pressure, mmHg | 135.00 [117.00-155.00] | 138.00 [123.00-159.00] | 0.001 |
| Heart rate, bpm | 82.00 [70.00-96.00] | 81.00 [70.00-95.00] | 0.914 |
| BUN, mg/dL | 30.00 [20.00-48.00] | 29.00 [21.00-52.00] | 0.333 |
| Creatinine, mg/dL | 1.40 [1.00-2.00] | 1.50 [1.10-2.18] | 0.059 |
| Sodium, mmol/L | 140.00 [136.00-142.00] | 139.00 [137.00-142.00] | 0.821 |
| Hospital length of stay, days | 5.24 [3.04-8.60] | 3.93 [1.55-7.09] | <0.001 |
| COPD history | 949/4,812 (19.7) | 143/696 (20.5) | 0.610 |
| Black race indicator | 1,125/4,812 (23.4) | 167/696 (24.0) | 0.720 |
| 30-day mortality | 317/4,812 (6.6) | 50/696 (7.2) | 0.556 |
| ICU admission within 24 h after ED disposition | 822/4,812 (17.1) | 174/696 (25.0) | <0.001 |
| Any ICU admission | 1,098/4,812 (22.8) | 197/696 (28.3) | 0.001 |
| In-hospital AKI | 1,373/4,810 (28.5) | 82/262 (31.3) | 0.337 |

| **B. Missingness of key variables** | | | | | | |
| --- | --- | --- | --- | --- | --- | --- |
| **Variable** | **Total missing, n** | **Total missing, %** | **Included missing, n** | **Included missing, %** | **Excluded missing, n** | **Excluded missing, %** |
| age_years | 0 | 0.0 | 0 | 0.0 | 0 | 0.0 |
| sbp_mmhg | 234 | 4.25 | 0 | 0.0 | 234 | 33.62 |
| heart_rate_bpm | 227 | 4.12 | 0 | 0.0 | 227 | 32.61 |
| bun_mg_dl | 447 | 8.12 | 0 | 0.0 | 447 | 64.22 |
| creatinine_mg_dl | 436 | 7.92 | 2 | 0.04 | 434 | 62.36 |
| sodium_mmol_l | 440 | 7.99 | 0 | 0.0 | 440 | 63.22 |
| copd_history | 0 | 0.0 | 0 | 0.0 | 0 | 0.0 |
| black | 0 | 0.0 | 0 | 0.0 | 0 | 0.0 |
| adhere_cart_group | 672 | 12.2 | 0 | 0.0 | 672 | 96.55 |
| gwtg_hf_score | 696 | 12.64 | 0 | 0.0 | 696 | 100.0 |
| death_30d | 0 | 0.0 | 0 | 0.0 | 0 | 0.0 |
| icu_within_24h | 0 | 0.0 | 0 | 0.0 | 0 | 0.0 |
| icu_anytime | 0 | 0.0 | 0 | 0.0 | 0 | 0.0 |
| aki_in_hospital | 436 | 7.92 | 2 | 0.04 | 434 | 62.36 |
| hospital_los_days | 0 | 0.0 | 0 | 0.0 | 0 | 0.0 |

| **C. Score missingness pattern** | | |
| --- | --- | --- |
| **Pattern** | **n** | **% of total** |
| ADHERE missing | 672 | 12.2 |
| GWTG-HF missing | 696 | 12.64 |
| Both ADHERE and GWTG-HF missing | 672 | 12.2 |
| ADHERE present but GWTG-HF missing | 24 | 0.44 |
| GWTG-HF present but ADHERE missing | 0 | 0 |
