# Supplementary material for: ADHERE CART versus GWTG-HF for 30-day mortality and intensive care outcomes in emergency department patients with heart failure: A retrospective cohort study (MIMIC-IV-ED)
Source: Medicine (Baltimore). 2026 May 22;105(21):e49037. doi: 10.1097/MD.0000000000049037 (PMC13200985; doi:10.1097/MD.0000000000049037)
Supplement: Supplementary file 4 [file medi-105-e49037-s004.docx]

| **Supplementary Table S4. Repeated-encounter distribution in the final analytic cohort** |
| --- |
| Final complete-case cohort included 4,812 encounters from 3,317 unique patients. |

| **A. Overall repeated-encounter summary** | | |
| --- | --- | --- |
| **Metric** | **Value** | **Percent** |
| Total encounters | 4812 | 100.0 |
| Unique patients | 3317 | 100.0 |
| Patients with 1 encounter | 2495 | 75.2 |
| Patients with >1 encounter | 822 | 24.8 |
| Encounters contributed by patients with >1 encounter | 2317 | 48.2 |

| **B. Collapsed distribution** | | | | |
| --- | --- | --- | --- | --- |
| **Encounter category** | **Number of patients** | **Total encounters contributed** | **Percent of patients** | **Percent of encounters** |
| 1 encounter | 2495 | 2495 | 75.2 | 51.8 |
| 2 encounters | 494 | 988 | 14.9 | 20.5 |
| ≥3 encounters | 328 | 1329 | 9.9 | 27.6 |

| **C. Exact distribution** | | | | |
| --- | --- | --- | --- | --- |
| **Encounters per patient** | **Number of patients** | **Percent of patients** | **Total encounters contributed** | **Percent of encounters** |
| 1 | 2495 | 75.2 | 2495 | 51.8 |
| 2 | 494 | 14.9 | 988 | 20.5 |
| 3 | 179 | 5.4 | 537 | 11.2 |
| 4 | 77 | 2.3 | 308 | 6.4 |
| 5 | 35 | 1.1 | 175 | 3.6 |
| 6 | 14 | 0.4 | 84 | 1.7 |
| 7 | 8 | 0.2 | 56 | 1.2 |
| 8 | 3 | 0.1 | 24 | 0.5 |
| 9 | 7 | 0.2 | 63 | 1.3 |
| 10 | 2 | 0.1 | 20 | 0.4 |
| 11 | 1 | 0 | 11 | 0.2 |
| 24 | 1 | 0 | 24 | 0.5 |
| 27 | 1 | 0 | 27 | 0.6 |
