# Supplementary material for: ADHERE CART versus GWTG-HF for 30-day mortality and intensive care outcomes in emergency department patients with heart failure: A retrospective cohort study (MIMIC-IV-ED)
Source: Medicine (Baltimore). 2026 May 22;105(21):e49037. doi: 10.1097/MD.0000000000049037 (PMC13200985; doi:10.1097/MD.0000000000049037)
Supplement: Supplementary file 5 [file medi-105-e49037-s005.docx]

| **Supplementary Table S5. First-encounter sensitivity analysis for 30-day mortality** |
| --- |
| First-encounter cohort was restricted to the first eligible encounter per patient. Values are unadjusted score-only models. |

| **A. Cohort counts** | | | | |
| --- | --- | --- | --- | --- |
| **Cohort** | **Encounters** | **Unique patients** | **30-day mortality events** | **30-day mortality, %** |
| Full encounter-level cohort | 4812 | 3317 | 317 | 6.6 |
| First-encounter cohort | 3317 | 3317 | 229 | 6.9 |

| **B. Primary outcome performance** | | | | | | | |
| --- | --- | --- | --- | --- | --- | --- | --- |
| **Cohort** | **Score** | **n** | **Events** | **Event rate, %** | **AUROC (95% CI)** | **OR (95% CI)** | **P value** |
| Full encounter-level cohort | ADHERE CART | 4812 | 317 | 6.6 | 0.644 (0.612-0.674) | 1.98 (1.74-2.26) | <0.001 |
| Full encounter-level cohort | GWTG-HF | 4812 | 317 | 6.6 | 0.748 (0.722-0.774) | 2.32 (2.08-2.60) | <0.001 |
| First-encounter cohort | ADHERE CART | 3317 | 229 | 6.9 | 0.641 (0.605-0.679) | 2.08 (1.77-2.44) | <0.001 |
| First-encounter cohort | GWTG-HF | 3317 | 229 | 6.9 | 0.747 (0.717-0.777) | 2.32 (2.03-2.64) | <0.001 |

| **C. AUROC difference, GWTG-HF minus ADHERE CART** | | | | | | | | |
| --- | --- | --- | --- | --- | --- | --- | --- | --- |
| **Cohort** | **Outcome** | **n** | **Events** | **ADHERE AUROC** | **GWTG-HF AUROC** | **AUROC difference** | **Difference 95% CI** | **P value** |
| Full encounter-level cohort | 30-day mortality | 4812 | 317 | 0.644 | 0.748 | 0.104 | 0.079-0.130 | <0.001 |
| First-encounter cohort | 30-day mortality | 3317 | 229 | 0.641 | 0.747 | 0.107 | 0.076-0.138 | <0.001 |
